# Expression analysis of *glyoxalase I* gene among patients of diabetic retinopathy

**DOI:** 10.12669/pjms.341.13839

**Published:** 2018

**Authors:** Aneela Rasul, Amir Rashid, Palvasha Waheed, Saleem Ahmed Khan

**Affiliations:** 1 Dr. Aneela Rasul, M Phil. Department of Biochemistry & Molecular Biology, Army Medical College, National University of Medical Sciences, Rawalpindi, Pakistan; 2 Dr. Amir Rashid, PhD. Department of Biochemistry & Molecular Biology, Army Medical College, National University of Medical Sciences, Rawalpindi, Pakistan; 3 Dr. Palvasha Waheed, PhD. Department of Biochemistry & Molecular Biology, Army Medical College, National University of Medical Sciences, Rawalpindi, Pakistan; 4 Dr. Saleem Ahmed Khan, FCPS, PhD. Department of Pathology, Army Medical College, National University of Medical Sciences, Rawalpindi, Pakistan

**Keywords:** Diabetic retinopathy, *Glyoxalase I*, Methylglyoxal

## Abstract

**Objectives:**

To study expression of *glyoxalase I* in patients of diabetic retinopathy.

**Methods:**

This cross-sectional comparative study was conducted at Centre for Research in Experimental and Applied Medicine (CREAM), Department of Biochemistry and Molecular Biology, Army Medical College, Rawalpindi in collaboration with Armed Forces Institute of Ophthalmology (AFIO) from January 2015 to November 2015. Sampling technique was non- probability purposive sampling. Total 60 subjects were enrolled in two groups. Group-I comprised 30 patients of diabetic retinopathy and Group-II of 30 normal healthy controls. Clinical and demographic data was collected and fasting venous blood samples (2 ml) were drawn. RNA was extracted and subjected to cDNA synthesis. Expression analysis for *glyoxalase I* was carried out and relative quantification done by double delta Ct method.

**Results:**

Mean age of the patients was 61.30 ±7.06 years and mean age of controls was 59.60 ± 6.43 years. There were 17 (56.7%) males and 13 (43.3%) females in Group-I while Group-II comprised 14 (46.7%) males and 16 (53.3%) females. There was down regulation of *glyoxalase I* among patients of diabetic retinopathy in comparison with controls when relative gene expression was calculated.

**Conclusion:**

Down regulation of *glyoxalase I* in patients of diabetic retinopathy suggests it to be a contributory factor in the development of disease.

## INTRODUCTION

Diabetes mellitus (DM) is a worldwide prevalent disease and the number of people suffering DM is increasing worldwide as well as in Pakistan because of aging, population growth, urbanization, and obesity. Statistics show that approximately 422 million people worldwide have DM in 2014 making the prevalence approximately 8.5%. DM is said to be a disease of complications and chronic complications include both microvascular and macrovascular complications. One frequently encountered complication is diabetic retinopathy (DR) which causes visual impairment of varying degrees and even blindness. The prevalence of retinopathy of any stage in patients of DM is 35% while that of proliferative diabetic retinopathy (PDR) is 7%.^1^

Various mechanisms have been proposed to explain the pathogenesis of diabetic complications. These include increased Protein C kinase activation, increased formation of advanced glycation end products (AGEs), accumulation of sorbitol via polyol pathway, reactive oxygen species (ROS) mediated cellular damage and increased flux through hexosamine pathway.^2^ A recent addition to this list is down regulation of *glyoxalase I* (GLO I) in chronic hyperglycemia.^3^

Hyperglycemia results in increased levels of triose phosphates, dihydroxyacetone phosphate (DHAP) and glyceraldehyde-3-phosphate (GA3P) in the cells leading to high flux of these triose phosphates to highly reactive methylglyoxal (MGO) formation. This abnormal accumulation of MGO and glyoxal is called dicarbonyl stress.^4^ MGO readily reacts with DNA, RNA and proteins, especially with arginine, to form advanced glycation end products (AGEs). MG-derived hydroimidazolone (MG-H1) is one of the most frequently formed AGEs.^5^

Glyoxalase system plays an important role in detoxification of MGO and other α-oxoaldehydes by converting them to the corresponding α-hydroxyacids. It comprises two enzymes, glyoxalase I (GLO I) and glyoxalase II (GLO II) with glutathione as a cofactor. This system detoxifies reactive metabolites accumulating during hyperglycemia.^6^ The substrates for GLO I are MGO, glyoxal and some other α-oxoaldehydes. Hemithioacetal is formed spontaneously from α-oxoaldehydes and GLO I catalyzes its isomerisation to S-2-hydroxyacylglutathione. The second enzyme in the system is GLO II, a thiolesterase that catalyzes the hydrolysis of *S*-D-lactoylglutathione to glutathione and D-lactic acid.^7^ The key enzyme of glyoxalase system is GLO I. It is expressed in cytosol of all the cells although its expression varies with age, type of tissue and health status of the individual^7^. Locus of *GLO I* is 6p21.2.

It is estimated that 99.7% of MGO is metabolized by glyoxalase system and only 0.3% is left to form glycation adducts.^3^ In diabetic patients, the flux of glucotriose to MGO formation increases two to four times depending upon the glycemic control but the rate of formation of MG and AGEs is disproportionately higher because of down regulation of *GLO I*. Insulin resistance further aggravates the diabetic complications. There are many reasons of this resistance. MGO mediated modification of insulin markedly decreases its action and is the major contributor of insulin dysfunction.^8^ Another important factor is AGEs mediated production of tumor necrosis factor-α (TNF-α), capable of blocking insulin signaling pathway.^9^ MGO is also capable of directly blocking insulin signaling pathway and preventing phosphorylation of protein kinase- B.^10^ MG-H1 has got very high affinity for receptor for AGEs (RAGE) and in DR there is RAGE dependent down regulation of *Glo I* that sets in a vicious cycle.^11^

Considering the high prevalence of diabetic retinopathy and diabetes mellitus among Pakistani population, this study was performed to analyze the expression of *GLO I* in DR. Glyoxalase system is under extensive study and *GLO I* inducers are being studied for prevention and treatment of diabetic complications.

## METHODS

This cross-sectional comparative study was conducted at Centre for Research in Experimental and Applied Medicine (CREAM), Department of Biochemistry and Molecular biology, Army Medical College, Rawalpindi from January 2015 to November 2015 in collaboration with Armed Forces Institute of Ophthalmology (AFIO). Study approval was granted by ethical review committee of Army Medical College, Rawalpindi. Total sample size was 60 (calculated by WHO calculator) divided into two groups. Diagnosed patients of proliferative diabetic retinopathy (PDR) by an ophthalmologist, between 40- 70 years of age were enrolled in Group-I from AFIO after seeking approval from ethical review committee. Patients of type 2 DM were enrolled only. For Group-II, age and gender matched normal healthy individuals were enrolled from general population. Patients having any co-morbidity or chronic illness and non-diabetic retinopathy were not included. Demographic and clinical data was collected for both the groups on a specifically designed Proforma. Fasting venous blood samples (2 ml) were collected after written informed consent. Total ribonucleic acid (RNA) was extracted from blood the same day after venous blood with drawl following the protocol provided by the kit manufacturer (Thermoscientific, USA) and stored at -80°C for downstream applications. Complementary deoxyribonucleic acid (cDNA) was synthesized from RNA by reverse transcriptase using revertaid first strand cDNA synthesis kit (Thermoscientific, USA).

Forward and reverse primers were designed for target gene (*GLO I*) and reference gene on the basis of available INFARI sequence on National Centre for Biotechnology Information (NCBI). *Glyceraldehyde phosphate dehydrogenase (GAPDH)* was the reference gene for normalization. The primer qualities were then evaluated using “Primer blast”. Sequence of both sets of primers is shown in Table-I.

**Table-I:** Sequence of primers for *GLO I* and *GAPDH.*

*Glyoxalase I*	
Forward primer (5´3´)	GGTGACTCCTCCCCTTG
Reverse primer (5´3´)	ACTCGTAGCATGGTCTGCTG
*GAPDH*	
Forward primer (5´3´)	GCTCTCTGCTCCTCCTGTTC
Reverse primer (5´3´)	TTCCCGTTCTCAGCCTTGAC

Polymerase chain reaction (PCR) conditions were optimized on Corbet Inc PCR machine. Synthesized cDNA was amplified by PCR followed by gel electrophoresis. After optimization, cDNA was subjected to amplification by real-time PCR (Cepheid smart cycler, USA) using Maxima SYBER Green PCR Master Mix by Thermoscientific, USA. Each sample was run in duplicates and cycle threshold (Ct) for amplification was noted down. Relative quantification of gene expression was done by ΔΔ Ct method.^12^

Data collected was entered on and analyzed by SPSS version 22. Normally distributed numerical data was expressed as mean ± standard deviation. Categorical data was expressed by percentages and frequency charts. Means were compared by t test.

## RESULTS

Mean age for Group-I was 61.30 ± 7.06 years and that of Group-II was 59.60 ± 6.43 years. There were 17 (56.7%) males and 13 (43.3%) females in Group-I while Group-II comprised 14(46.7%) males and 16(53.3%) females. Mean duration of DM in Group-I was 14.33 ± 5.49 years. Mean fasting blood glucose for Group-I was 10.75 ± 2.8 mmol/L and for Group-II, 4.8 ± 0.5 mmol/L at a highly significant p value of < 0.001. A significant difference in the means of HbA1c was noted down. The mean percentage of HbA1c among Group-I was 7.27 ± 0.82 while its value for Group-II was 5.03 ± 0.57.

**Fig. 1:**
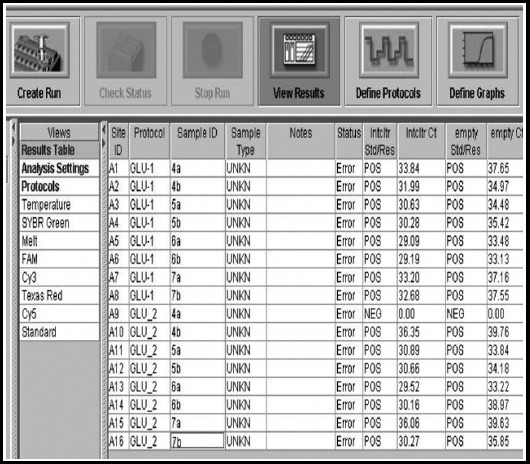
Ct values for *Glyoxalase I* of Group-I, an image of Real time PCR.

There was low abundance of *GLO I* in Group-I as the Ct values were in the range of 30-38. Ct values of Group-II were lower when mean values of both the groups were compared. Mean Ct values for *GAPDH* of both the groups were almost same. Mean Ct values of *GLO I* were significantly higher (p < 0.0001) in Group-I versus Group-II when compared by independent t test. Image of Ct values of Group-I for gene of interest is shown in Fig.1.

*GLO I* expression was found to be down regulated among Group-I compared with Group-II when calculated by double delta Ct method of relative quantification. The results are shown in Table-II.

**Fig. 2:**
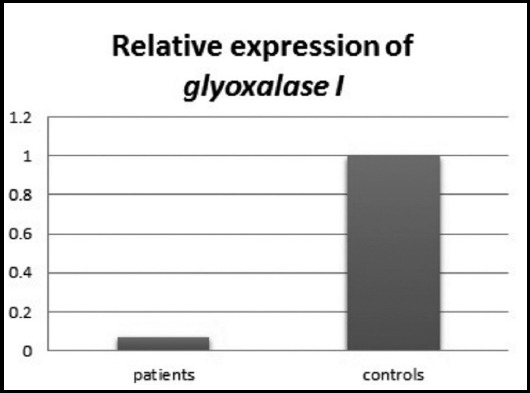
Relative expression of GLO I showing down regulation in patients compared with controls.

**Table-II:** Showing mean Ct, Δ CT, ΔΔ CT and fold difference of expression of GLO I in Group-I and Group-II.

	Mean CT Glo I	Mean CT GAPDH	Δ CT	ΔΔ CT	2^- ΔΔ CT^
Group-I	31.81 ± 2.49	21.17 ± 1.75	10.64	4.32	- 0.0698
Group-II	27.75 ± 2.79	21.43 ± 1.94	6.32		

## DISCUSSION

There was down regulation of *Glo I* among DR patients in comparison with controls in our study. Genomic study of human *Glo I* revealed that there is an insulin response element (IRE) in the gene and deficiency of insulin in DM leads to its down regulation.^13^ Glyoxalase system is under extensive study and results from other studies also reported down regulation of this system in diabetes especially where complications were reported.^14^

Giacco et al established in their study that *GLO I* knockdown in nondiabetic mice results in increased concentration of MGO and increased oxidative stresss. On the contrary their study on diabetic mice revealed that over expression of *GLO I* provide protection from oxidative stress and diabetic complications despite chronic hyperglycemia.^15^ Their study demonstrated that variations in MGO detoxification capacity determine the susceptibility to diabetic complications.^15^

There are some other ways in which GLO I is protective against hyperglycemia induced damage. Xue et al established that *GLO I* over expression is related with prevention of increased synthesis of ROS, certain inflammatory mediators like S100A12, S100A8 and high-mobility box-1 protein and decreased expression of RAGE.^16^

In addition to down regulation of *GLO I* in prolonged hyperglycemia, there also exists decreased efficiency of glyoxalase enzyme system. This occurs because of decreased flux through pentose phosphate shunt thus depleting the cells of NADPH. The result is decreased regeneration of GSH which is essential for efficient functioning of glyoxalase system.^17^ Down regulation and decreased efficiency of GLO I lead to accumulation of MGO which is 20,000 times more reactive than glucose to form AGEs.^18^

Intracellular MGO levels are regulated by aldose reductase (AR) pathway in addition to glyoxalase mediated detoxification. MGO is a substrate for AR and reduced form of glutathione (GSH) is also required for enzymatic activity.^19^ In the tissues with high GSH and low AR, glyoxalase enzyme system becomes the major pathway for detoxification of MGO. With the exception of renal glomeruli, all the human tissue including retina are mainly dependent upon glyoxalase system for MGO detoxification. Berner et al reported that MGO related retinal damage can be prevented by over expression of *Glo I*. Raised GLO I levels provide protection by minimizing the MGO derived AGEs synthesis.^20^

In DR visual impairment starts during proliferative stage when there is angiogenesis. *GLO I* has got a key role in suppression of AGEs formation and it's over expression is capable of reversal of angiogenesis and AGEs synthesis in endothelial cells.^4^ *GLO I* inducers are being studied and in future may be used as therapeutic agents for prevention and treatment of diabetic complications.

### Limitations of the study

Limited financial resources were the major constraint for the study and sample size for expression analysis was small because of it. Study participants were enrolled from a narrow range of ethnicity and a single center. A third group of diabetic patients without complications should be added for better comparison and analysis.

## CONCLUSION

The study concluded that there is down regulation of *GLO I* among patients of DR when compared with normal healthy controls and down regulation of *GLO I* plays an important role in the development of diabetic complications including DR along with various other mechanisms.

### Authors' Contributions

***AS:*** Data collection, Data analysis and interpretation, drafting the article.

***AR and PW:*** Conception and design of the work, Critical revision of the article.

***SAK:*** Did review and final approval of the version to be published.
